# A qualitative survey on factors affecting depression and anxiety in patients with rheumatoid arthritis: a cross-sectional study in Syria

**DOI:** 10.1038/s41598-024-61523-3

**Published:** 2024-05-20

**Authors:** Fater A. Khadour, Younes A. Khadour, Bashar M. Ebrahem

**Affiliations:** 1 Department of Rehabilitation, Tongji Hospital, Tongji Medical College, Huazhong University of Science and Technology, Wuhan, 430030 China; 2 Department of Physical Therapy, Cairo University, Cairo, 11835 Egypt; 3 Department of Rehabilitation, Faculty of Medicine, Al Baath University, Homs, Syria

**Keywords:** Anxiety, Depression, Rheumatoid arthritis, Disease activity, Outcomes research, Medical research, Rheumatology, Rheumatic diseases

## Abstract

Depression and anxiety often coexist with rheumatoid arthritis (RA) and affect the course of the disease. These mental health conditions can be overlooked or underdiagnosed in people with RA. There is conflicting evidence in previous studies regarding this topic, indicating that further research is necessary to provide a thorough understanding of the relationship between anxiety, depression, and RA. This study aims to determine the factors correlated with depression and anxiety symptoms in RA patients by evaluating disease activity at the same time. This cross-sectional study was conducted at four outpatient rehabilitation centers in four Syrian provinces: Damascus, Homs, Hama, and Latakia. The study included RA patients who attended the RA department of rehabilitation centers from January 1 to June 31, 2023. RA patients who presented at a rheumatology clinic were selected consecutively. RA patients were included in the study in accordance with the ACR/EULAR classification criteria, disease activity was assessed by disease activity score based on the 28-joint count (DAS28), and patients with DAS28 > 2.6 were considered to have active RA. The demographic data, as well as disease duration, educational status, Disease Activity Score with 28-joint counts (DAS28), health assessment questionnaire (HAQ) score, and the hospital anxiety and depression scale (HADS), were the parameters used in the analysis. Two hundred and twelve patients (female, 75%) with a mean age of 49.3 ± 13.1 years and a mean disease duration of 8.3 ± 6.9 years were studied. Depression was diagnosed in 79 (37.3%) patients and anxiety in 36 (16.9%) patients. Patients with depression and/or anxiety had higher HAQ and DAS28 scores compared to other RA patients. Blue-collar workers exhibited a higher prevalence of anxiety, whereas females, housewives, and individuals with lower educational attainment demonstrated a higher prevalence of depression. The current study found high rates of anxiety and depression in RA patients, highlighting the significant burden of these mental health conditions compared to the general population. It is essential for healthcare providers not to overlook the importance of psychiatric evaluations, mental health assessments, and physical examinations of RA patients.

## Introduction

Rheumatoid arthritis (RA) is a chronic autoimmune disease primarily characterized by joint inflammation and damage. While its primary symptoms are related to joint pain, swelling, and stiffness, RA can also have systemic effects that extend beyond the joints. These systemic effects can include fatigue, sleep disturbances, and mood changes. This overlap can result in RA patients experiencing symptoms similar to those observed in individuals with depression^1^. Anxiety is also prevalent among individuals with RA, with approximately 20% of patients undergoing anxiety^2^. Another study has indicated that nearly 30% of RA patients experienced symptoms of depression during developing the disease^3^.

Patients diagnosed with RA who present with comorbid anxiety and depression symptoms tend to exhibit worse health outcomes, including poor medication adherence^4^, suboptimal response to treatment^4^, elevated medical costs^5^, increased mortality^6^, and diminished quality of life^7^. As a result, it is critical to investigate the risk factors for anxiety and depression symptoms in RA patients and to incorporate psychological management into their medical care. Several research studies have been carried out to improve RA control and prevention^8–11^.

According to a meta-analysis, depression was present in 17% of RA patients^8^. Additionally, another meta-analysis that included 10 cohort studies revealed that RA patients are at a significantly higher risk for anxiety than individuals without RA, with an odds ratio of 1.20 (95% confidence interval: 1.03–1.39)^9^. Another study of RA patients revealed a prevalence of 38.4% for patient-reported depression or anxiety, but only 17.7% of patients were diagnosed with depression or depression by their physicians. According to the same study^10^, patients with anxiety or depression had significantly higher levels of treatment dissatisfaction and impaired job and everyday activity.

The incidence of anxiety and depression in RA patients and their relationship with disease severity has varied among researchers. These inconsistencies could be related to differences in study populations, diagnostic criteria applied, the severity of depression or anxiety evaluated, and the distribution of associated factors in the general population. Furthermore, disease activity in RA patients, which reflects the aforementioned conditions, may contribute to developing depression and anxiety symptoms. However, study findings on the correlation between disease activity and anxiety and depression are inconsistent. Some studies have suggested a positive association between RA disease activity and anxiety and depression symptoms^12,13^, whereas others have not identified an association^14^.

Depression and rheumatoid arthritis (RA) demonstrate an interconnected relationship, as both conditions are linked to inflammation. Numerous studies have explored the impact of depression and inflammation on pain perception in RA^12^. These studies highlight that depression is frequently associated with more severe RA and unfavorable outcomes^6^. Additionally, symptoms of depression and anxiety correlate with subjective aspects of disease activity, reducing the likelihood of RA remission and influencing treatment decisions.

Research has shown that individuals with RA may experience higher rates of anxiety and depression compared to the general population. The chronic pain, physical limitations, and unpredictable nature of the disease can contribute to increased psychological distress. Additionally, the inflammatory processes involved in RA can have an impact on the central nervous system and neurotransmitter function, potentially contributing to the development or exacerbation of anxiety and depression^15,16^. It's important to note that anxiety and depression in RA can be influenced by various factors, including disease activity, pain levels, functional limitations, and socioeconomic factors. Therefore, it is crucial to address both the physical and psychological aspects of RA to provide comprehensive care for individuals with the condition^17,18^.

However, the prevalence of depression and anxiety among RA patients and their association with RA severity exhibit variability across different studies. This variability can be attributed to various factors, including the characteristics of the study population, criteria used for diagnosing and assessing the severity of depression or anxiety, methods employed for measuring RA activity, and the distribution of factors associated with depression or anxiety within the general population. Moreover, the coexistence of depression and anxiety with RA often goes undiagnosed or unrecognized due to the overlap in symptoms between these conditions and RA itself. In light of these challenges, the current study aims to determine the factors associated with depression and anxiety symptoms in RA patients by evaluating disease activity at the same time.

## Methods

### Patients and setting

This cross-sectional study was conducted at four outpatient rehabilitation centers in four Syrian provinces: Damascus, Homs, Hama, and Latakia. The study included RA patients who attended the RA department of rehabilitation centers from January 1 to June 31, 2023. This study included all the patients who met the 2010 American College of Rheumatology/European League Against Rheumatism classification criteria^19^,while depression and anxiety were diagnosed using the hospital anxiety and depression scale (HADS)^20^, a 14-item questionnaire with seven subscales for anxiety and depression symptoms. Each item is scored on a scale of 0–3, the total score range for each condition is 0–21. Scores of 0–7 indicate no or few anxiety or depression symptoms, 8–10 indicate mild anxiety or depression, and ≥ 11 indicate severe anxiety or depression*.* The Arabic version of the HADS has been widely used to screen patients with a variety of diseases and has been previously validated for use with the Arabic population^21^. In this study, we defined anxiety and depression as a HADS anxiety score ≥ 8 and a HADS depression score ≥ 8, respectively^21^.

Patient data has been obtained, including age, gender, marital status, employment position, BMI, disease duration, comorbidities, and medication use. The DAS28-ESR was used to assess disease activity, which is based on a 28-joint assessment; 28 tender joint counts (TJC), 28 swollen joint counts (SJC); and the patient global assessment (PtGA)^22^. [R1] The HAQ score was used to evaluate functional status^23^. Pain was evaluated using either the visual analogue scale (VAS), on which items were scored from 0 (no pain) to 100 (maximum pain)^24^.

This study included all patients who met the ACR/EULAR classification criteria, aged between 18 and 85 years and were willing to participate and provide informed consent. Any patient has a history of other autoimmune or inflammatory conditions (e.g. systemic lupus erythematosus, psoriatic arthritis), severe cognitive impairment or neurological disorders that may hinder accurate reporting of depression and anxiety symptoms, pregnant or lactating women, as hormonal changes during these periods can affect mood and anxiety levels, in addition to any patients has a history of psychological disorders (e.g. bipolar disorder, schizophrenia) or had coexisting chronic conditions such as chronic low back pain, chronic non-RA musculoskeletal diseases, cardiovascular disease, cerebrovascular diseases, and gastrointestinal diseases, were excluded from the study.

The Ethical Committee approved this study in the Al Baath University Institutional Review Board Consent Letter – IRB 2023168-S and all procedures were conducted under the ethical principles outlined in the 1964 Declaration of Helsinki and its subsequent revisions. Patients were informed of the study's purpose and procedures. In addition, written informed consent to participate in this study was provided by the participants.

### Statistical analysis

The statistical analyses were performed with the assistance of version 23.0 of the SPSS for Windows software package. The data was evaluated using descriptive statistics such as means, standard deviations, and frequencies. Categorical data was measured using the chi-square test, while continuous variables were computed using Student’s *t*-test or the Mann–Whitney *U* test. Multivariate logistic regression analyses were used to determine the relationship between clinical and demographic factors and anxiety depression or anxiety among rheumatoid arthritis patients. The results were presented as odds ratios with 95% confidence intervals. A p-value less than 0.05 was considered statistically significant.

### Ethics approval and consent to participate

The Ethical Committee approved this study in the Al Baath University Institutional Review Board Consent Letter – IRB 2023168-S and all procedures were conducted under the ethical principles outlined in the 1964 Declaration of Helsinki and its subsequent revisions. Patients were informed of the study's purpose and procedures. In addition, written informed consent to participate in this study was provided by the participants.

## Results

This study included a cohort of 212 patients diagnosed with RA. The patients had a mean age (SD) of 49.3 ± 13.1 years (ranging from 20 to 73 years), and the average duration of the disease was 8.3 ± 6.9 years (ranging from 2 to 46 years). Among the participants, 70% were female. The mean DAS28 (SD) was 2.6 ± 1.2, and the mean HAQ score was 1.08 ± 1.2.

Tables 1 and 2 provides an overview of the descriptive statistics for various variables, including age, BMI, disease duration, DAS28, HAQ, and HADS scores, as well as information on gender, marital and working status, education level, comorbidities, and medications utilized. Based on the Arabic validation scores of the hospital anxiety and depression scale (HADS), anxiety symptoms were present in 16.9% of the patients, while depression symptoms were detected in 37.3% of the participants.

**Table 1:** Demographic and clinical characteristics.

Variable	Mean ± SD	Range
Age (years)	49.3 ± 13.1	20–73
BMI (kg/m^2^)	27.7 ± 3.31	17.3–47.1
Disease duration (years)	8.3 ± 6.9	2–46
DAS28	2.6 ± 1.2	0.7–7.1
TJC	0.87 ± 0.5	0.77–0.92
SJC	0.84 ± 0.6	0.73–0.96
PtGA	28.4 ± 13.6	26.5–32.3
Pain VAS	26.4 ± 11.7	24.6–29.6
HAQ	1.08 ± 1.2	0–3
HADS – anxiety	7.5 ± 4.3	0.7–7.1
HADS – depression	1.2 ± 1.1	0–3

	Number (%)	
Comorbidities		
None	133 (62.7)	
Present	79 (37.3)	
Drugs		
csDMARD	153 (72.2)	
bDMARD	59 (27.8)	

*BMI* body mass index, *bDMARD* biological disease-modifying antirheumatic drug, *csDMARD* conventional synthetic disease-modifying antirheumatic drug, *DAS28* disease activity score with 28-joint counts, *TJC* tender joint count, *SJC* swollen joint count, *PtGA* patient global assessment, *HADS* hospital anxiety depression scale, *HAQ* health assessment questionnaire.

**Table 2:** The χ^2^ test results of demographic characteristics between patients with and without anxiety and depression.

Demographic characteristics between patients with and without anxiety						
Sex		Women	Men	Total		p = 0.328
Anxiety −	n (%)	124 (83.2)	52 (82.5)	176 (83.0)		
Anxiety +	n (%)	25 (16.8)	11 (17.5)	36 (16.9)		
Total	n (%)	149 (100)	63 (100)	212 (100)		
Marital status		Married	Single	Total		p = 0.426
Anxiety −	n (%)	139 (92.0)	37 (60.6)	176 (83.0)		
Anxiety +	n (%)	12 (8.0)	24 (39.3)	36 (16.9)		
Total	n (%)	151 (100)	61 (100)	212 (100)		
Education						p = 0.349
		Uneducated	Primary	High school	University	Total
Anxiety −	n (%)	24 (75.0)	74 (80.4)	41 (87.2)	37 (90.2)	176 (83.0)
Anxiety +	n (%)	8 (25.0)	18 (19.6)	6 (12.8)	4 (9.8)	36 (16.9)
Total	n (%)	32 (100)	92 (100)	47 (100)	41 (100)	212 (100)
Working status						**p = 0.033**
		Retired	Housewife	White-collar	Blue-collar	Total
Anxiety −	n (%)	42 (89.4)	85 (83.3)	11 (57.9)	8 (57.1)	176 (83.0)
Anxiety +	n (%)	5 (10.6)	17 (16.6)	8 (42.1)	6 (42.9)	36 (16.9)
Total	n (%)	47 (100)	102 (100)	19 (100)	14 (100)	212 (100)
Comorbidities		None	Present	Total		p = 0.751
Anxiety −	n (%)	110 (82.7)	66 (83.5)	176 (83.0)		
Anxiety +	n (%)	23 (17.3)	13 (16.5)	36 (16.9)		
Total	n (%)	133 (62.7)	79 (37.3)	212 (100)		
Drugs		DMARD	Biological	Total		p = 0.472
Anxiety −	n (%)	136 (88.9)	40 (67.8)	176 (83.0)		
Anxiety +	n (%)	17 (11.1)	19 (32.2)	36 (16.9)		
Total	n (%)	153 (72.2)	59 (27.8)	212 (100)		

Demographic characteristics between patients with and without depression						
Sex		Women	Men	Total		p = 0.371
Depression −	n (%)	92 (61.7)	41 (82.2)	133 (62.7)		
Depression +	n (%)	57 (38.2)	22 (34.9)	79 (37.3)		
Total	n (%)	149 (100)	63 (100)	212 (100)		
Marital status		Married	Single	Total		p = 0.163
Depression −	n (%)	105 (49.5)	50 (81.9)	133 (62.7)		
Depression +	n (%)	46 (30.5)	11 (18.1)	79 (37.3)		
Total	n (%)	151 (100)	61 (100)	212 (100)		
Education						p = 0.358
		Uneducated	Primary	High school	University	Total
Depression −	n (%)	19 (60.8)	49 (53.4)	32 (68.1)	33 (80.5)	133 (62.7)
Depression +	n (%)	13 (40.6)	43 (46.7)	15 (31.9)	8 (19.5)	79 (37.3)
Total	n (%)	32 (100)	92 (100)	47 (100)	41 (100)	212 (100)
Working status						p = 0.042
		Retired	Housewife	White-collar	Blue-collar	Total
Depression −	n (%)	42 (89.4)	71 (69.6)	15 (78.9)	8 (57.1)	133 (62.7)
Depression +	n (%)	5 (10.6)	31 (30.4)	4 (21.1)	6 (42.9)	79 (37.3)
Total	n (%)	47 (100)	102 (100)	19 (100)	14 (100)	212 (100)
Comorbidities		None	Present	Total		p = 0.641
Anxiety −	n (%)	80 (60.2)	53 (67.1)	133 (62.7)		
Anxiety +	n (%)	53 (39.8)	26 (32.9)	79 (37.3)		
Total	n (%)	133 (62.7)	79 (37.3)	212 (100)		
Drugs		DMARD	Biological	Total		p = 0.431
Anxiety −	n (%)	96 (62.7)	37 (62.7)	133 (62.7)		
Anxiety +	n (%)	57 (37.3)	22 (37.3)	79 (37.3)		
Total	n (%)	153 (72.2)	59 (27.8)	212 (100)		

Significant values are in bold.

Tables 2 comprehensively compare various factors, including gender, marital and employment status, education level, comorbidities, and medication usage, among patients with and without anxiety and depression. The results revealed statistically significant differences in the working status between patients with and without anxiety and depression (p = 0.033, p = 0.042), respectively. Additionally, significant differences were observed in terms of sex and working status between patients with depression and those without depression (p < 0.05). Of particular note, it was found that anxiety levels were considerably higher in individuals employed in blue-collar occupations compared to retired patients. The prevalence of depression was significantly higher in women compared to men. Additionally, it was found to be more prevalent among patients with lower levels of education (uneducated) compared to those with a high school or university education. Furthermore, housewives had a higher prevalence of depression compared to retired patients. 

Regarding medication usage, a comparison was made between users of bDMARD and csDMARD. The analysis revealed no statistically significant difference in the levels of anxiety and depression between these two groups of medication users. This information is detailed in Table 2.

Table 3 presents the comparisons of BMI, age, duration of disease, HAQ, and DAS28 scores between different groups. It was observed that patients with both anxiety and depression had significantly higher DAS28 and HAQ scores compared to patients without depression and anxiety (p < 0.05), indicating higher disease activity and worse functional status.

**Table 3:** Comparison of demographic and clinical characteristics between patients with and without anxiety and depression.

Clinical characteristics between patients with and without anxiety			
Variable	Anxiety −	Anxiety +	p-value
Age	49.9 ± 11.3	51.3 ± 12.5	0.317
Body mass index	28.3 ± 3.9	29.7 ± 4.8	0.692
Disease duration	10.2 ± 7.2	11.3 ± 9.3	0.212
DAS28	2.4 ± 1.2	3.8 ± 1.1	0.032
TJC	0.79 ± 0.4	0.81 ± 0.5	0.311
SJC	0.80 ± 0.6	0.82 ± 0.4	0.082
PtGA	29.2 ± 12.7	28.9 ± 14.7	0.113
Pain VAS	28.3 ± 10.9	27.4 ± 11.5	0.105
HAQ	0.7 ± 1.3	1.4 ± 1.1	< 0.001

Clinical characteristics between patients with and without depression			
Variable	Depression −	Depression +	p-value
Age	52.7 ± 11.7	53.2 ± 11.2	0.411
Body mass index	28.2 ± 4.3	28.7 ± 4.9	0.523
Disease duration	11.2 ± 8.3	10.6 ± 9.2	0.211
DAS28	3.1 ± 1.7	2.7 ± 1.0	0.021
TJC	0.85 ± 0.4	0.86 ± 0.6	0.044
SJC	0.85 ± 0.6	0.84 ± 0.4	0.125
PtGA	26.7 ± 11.4	27.4 ± 12.4	0.061
Pain VAS	26.2 ± 12.3	27.6 ± 11.8	0.092
HAQ	0.8 ± 1.3	1.8 ± 1.7	< 0.001

Statistical significance was calculated using the Mann–Whitney *U* test.

*DAS28* disease activity score with 28-joint counts, *TJC* tender joint count, *SJC* swollen joint count, *PtGA* patient global assessment, *HAQ* health assessment questionnaire, *VAS* visual analog scales.

Table 4 shows the multivariate logistic regression analysis results with anxiety and depression as dependent variables. The HAQ score and DAS28 were both significantly associated with anxiety (OR = 1.09, 95% CI 0.94–1.05, p = 0.012) (OR = 1.44, 95% CI 0.95–1.87, p = 0.041) and depression (OR = 1.23, 95% CI 1.25–2.87, p = 0.032) (OR = 1.43, 95% CI 1.03–1.67).

**Table 4:** Multivariate analysis for demographic and clinical factors associated with anxiety and depression in patients with rheumatoid arthritis.

Variable	Anxiety		Depression	
Factors	OR (95% Cl)	p-value	OR (95% Cl)	p-value
Age	1.04 (0.94–1.12)	0.871	1.12 (0.91–1.24)	0.265
Body mass index	1.11 (0.96–1.13)	0.654	1.08 (0.87–1.14)	0.354
Disease duration	0.89 (088–1.04)	0.321	1.11 (0.97–1.21)	0.698
DAS28	1.44 (0.95–1.87)	0.041	1.43 (1.03–1.67)	0.137
TJC	1.89 (0.91–1.43)	0.621	1.79 (0.81–1.82)	0.172
SJC	1.01 (0.82–1.63)	0.832	1.21 (0.71–1.53)	0.134
PtGA	0.67 (0.63–1.31)	0.271	1.09 (0.84–1.03)	0.083
Pain VAS	2.12 (0.75–1.63)	0.214	1.74 (0.48–1.58)	0.072
HAQ	1.09 (0.94–1.05)	0.012	1.23 (1.25–2.87)	0.032
Sex	1.56 (0.65–7.11)	0.421	2.37 (1.05–7.09)	0.226
Marital status	1.13 (0.54–2.98)	0.623	0.45 (0.76–1.26)	0.432
Education				
Uneducated	1.02 (0.67–3.12)	0.432		0.241
Primary school	1.06 (0.52–3.25)	0.944	0.98 (0.87–2.98)	0.872
High school	1.17 (0.43–3.04)	0.831	0.76 (0.22–1.09)	0.432
University	0.43 (0.35–1.23)	0.311	0.44 (0.19–0.89)	0.434
Working status				
Retired		0.732		0.421
Housewife	1.43 (0.67–4.57)	0.582		0.743
White-collar worker	3.22 (0.53–5.69)	0.231	4.66 (0.98–10.87)	0.321
Blue-collar worker	3.65 (0.43–10.21)	0.265	2.78 (1.02–9.09)	0.243
Comorbidities	2.54 (0.78–3.89)	0.742	0.95 (0.87–1.76)	0.242
Drugs	2.76 (0.54–5.43)	0.327	0.99 (0.76–1.98)	0.287

*OR* (95% CI) odds ratios (95% confidence intervals). *DAS28* disease activity score with 28-joint counts, *HAQ* health assessment questionnaire.

## Discussion

RA is a chronic autoimmune disease that primarily affects the joints. It is characterized by inflammation of the synovial lining in multiple joints, leading to joint pain, swelling, stiffness, and progressive joint damage. RA is classified as an inflammatory type of arthritis. RA can also present with extra-articular manifestations, meaning it can affect other organs and systems in the body. These extra-articular manifestations can include symptoms such as depression, fatigue, and sleep disturbance^25,26^. This study aimed to determine the frequency and the factors affecting depression and anxiety in patients with RA.

In this study, depression was determined in 37.3% of the patients, and anxiety in 16.9%. Patients with depression and anxiety had significantly higher DAS28-ESR and HAQ scores than those without depression and anxiety. Depression was determined at a higher rate in females, patients with a low level of education, and housewives, while a university education level was associated with a reduced risk of depression. Anxiety was determined at higher rates in blue-collar workers. Our results are consistent with a study conducted by Altan et al.^27^ reported a depression rate of 44% and an anxiety rate of 38% in patients with RA, and a study conducted by Isık et al.^28^ that used the HADS-A and HADS-D scales and found anxiety and depression rates of 41.5% and 13.4% in patients with RA, On contract, in a systematic review of 21 studies that included 4,447 RA patients, found a prevalence of depression of 48%among RA patients^29^.

Different rates of depression and anxiety have been recorded in various research, and these disparities have been associated with factors such as study design, scales employed, and a probable relationship with geography and social and economic status^29^. In a study of Brazilian patients with RA, depression was more prevalent among Brazilians and high disease activity is associated with depression^30^. In another study conducted in Italy among RA patients, depression was detected in 14.3%. and it found a substantial rise in the risk of depression with male sex, a high HAQ score, patient global evaluation, and the use of antidepressants^31^. The results of this study are not consistent with previous studies, where sex, a high HAQ score, age, BMI, and disease duration do not show the risk of depression and anxiety, in return, it found that the work status has a significant increase in the risk of depression and anxiety among RA patients.

It is accepted that there is a two-way relationship between RA and depression^20,29^. Depression is seen more in RA patients, and there has been found to be an increased risk of RA development in individuals with depression. There are increased proinflammatory cytokines in depression similar to in RA, and these cytokines are reduced with antidepressant treatment^8^. In patients with severe depressive disorder, the risk of developing RA is increased by 38% compared to the normal population and the risk of RA development has been reported to be reduced in those using antidepressants compared to non-users^32^, some anti-cytokine treatments used in RA have been found to affect depression positively^33^.

In a study by Ng et al.^34^, anxiety and depression were strongly associated with DAS28-ESR. The study also found that depression was significantly lower in patients using etanercept, and these results are consistent with our study where anxiety and depression were associated with DAS28-ESR (p = 0.032, p = 0.021), respectively.

It is necessary to highlight the importance of the impact of depression and anxiety on the management and outcomes of rheumatoid arthritis. Understanding the association between mental health conditions and disease activity can aid in developing comprehensive treatment approaches for individuals with rheumatoid arthritis, wherein a study conducted by Matcham et al.^35^ on 18,421 RA patients receiving biological treatment revealed that the response to treatment in the first year was reduced by 20–40% when depression was present at the beginning of the treatment. These results suggest that depression can have a negative impact on the effectiveness of biological treatment in RA patients. In another study by Fragoulis et al.^36^, which involved 848 early RA patients, anxiety was reported to be 19.0%, while depression was 12.2%. The study also identified a relationship between depression and anxiety, disease activity, and poor functional outcomes in patients with early rheumatoid arthritis.

A low socioeconomic status, female sex, young age, and functional limitations have been reported to be factors associated with depression in RA patients^34^. Depression is generally associated with the severe form of RA^35^. In a meta-analysis, Zhang et al.^33^ determined higher disease activity and lower quality of life in RA patients with depression compared to those without depression^37^. In addition, Watad et al.^38^ found higher levels of anxiety in RA patients compared to a control group, and low socioeconomic status was reported to be an independent factor associated with anxiety. In another study, low socioeconomic status and high DAS28 scores were determined to be associated with anxiety^36^. Our results are in line with previous studies which showed that individuals diagnosed with RA who also experienced depression and anxiety displayed higher levels of disease activity and lower quality of life compared to RA patients without, but no difference was determined concerning pain.

In our study, when bDMARD and csDMARD users were compared, no statistically significant difference was found in terms of anxiety and depression. However, we noticed a substantial variation in patients' DAS28 and HAQ scores and the presence of depression and anxiety. Similarly, in another study, bDMARDs and csDMARDs were not superior in depression^36^. More research is needed to investigate the impact of bDMARDs on anxiety and depression. A study of 464 RA patients found that depression was associated with the global health score, while anxiety was associated with being married and having a functional disability^39^. In another study, it was reported that the presence of anxiety and depression in patients with RA can cause suicide and diminished quality of life and can worsen the prognosis of RA^40^. The study had some limitations, including a relatively small sample size, and a cross-sectional design. Additionally, there was no control group in the study, and the patients' social and economic situation was not investigated. Since in just four rehabilitation centers, the results may not be generalizable to all RA patients.

## Conclusion

Anxiety and depression are highly prevalent among (RA) patients, and it is important to consider that this may impact the patients’ response to treatment, prognosis, and even mortality. Therefore, it is recommended to collaborate with the psychiatry department in managing these cases.

## Data Availability

The original contributions presented in the study are included in the article, further inquiries can be directed to the corresponding author.
